# Prognostic Role of MicroRNA-210 in Various Carcinomas: A Systematic Review and Meta-Analysis

**DOI:** 10.1155/2014/106197

**Published:** 2014-01-23

**Authors:** Minmin Li, Xuelei Ma, Mei Li, Binglan Zhang, Juan Huang, Lei Liu, Yuquan Wei

**Affiliations:** The Department of Medical Oncology, Cancer Center, State Key Laboratory of Biotherapy, West China Hospital, Sichuan University, Chengdu 610041, China

## Abstract

*Objective*. Many studies have shown that microRNAs (miRNAs) could play a potential role as prognostic biomarkers of tumors. The aim of this study is to summarize the global predicting role of microRNA-210 (miR-210) for survival in patients with a variety of carcinomas. *Methods*. Relevant literature was identified using PubMed and the information in eligible studies has been extracted. Then meta-analysis of hazard ratio (HR) was performed to evaluate the prognostic role of the miR-210 in different tumors. *Results*. This meta-analysis included 9 published studies dealing with various carcinomas. For recurrence free survival or disease free survival (RFS/DFS), the combined hazard ratio (HR) and 95% confidence interval (95% CI) of higher miR-210 expression were 2.47 [1.36, 4.46], which could significantly predict poor survival in general carcinomas. MicroRNA-210 was also a significant predictor for overall survival (OS), metastasis free survival or distant relapse free survival (MFS/DRFS), and disease specific survival (DSS). Importantly, subgroup analysis suggested that higher expression of miR-210 correlated with worse RFS/DFS, OS, and MFS/DRFS, especially in breast cancer, which were 3.36 [2.30, 4.93], 3.29 [1.65, 6.58], and 2.85 [1.76, 4.62] separately. *Conclusion*. Our studies suggested that microRNA-210 could predict the outcome of patients with varieties of tumors, especially in breast cancers.

## 1. Introduction

The discovery of the lin-4 small noncoding RNA in *C. elegans* in 1993 [1] initiated research focused on the cellular function of microRNAs (miRNAs). MicroRNAs (miRNAs), which are approximately 22-nucleotide long, single stranded, regulate gene expression at posttranscriptional levels. MiRNAs exert their regulatory effect by binding the 3′-UTR of their target mRNA and inhibiting target gene translation to protein [2]. Therefore, a specific miRNA may simultaneously regulate multiple targets, while a single target can be regulated by multiple miRNAs [3]. Furthermore, upstream regulation of a given miRNA can involve multiple regulators at different steps of miRNA biogenesis. Thus, miRNAs take part in crucial biological processes such as differentiation, proliferation, and apoptosis [3].

In 2002, two miRNAs, miR-15a and miR-16-1, were first revealed to be downregulated in patients with chronic lymphocytic leukemia [4]. Since then, for the past few years, numerous studies have demonstrated an involvement of miRNAs in tumor development and progression. Dysregulated miRNAs have been reported in various human cancers [5–9], and the expression levels of some miRNAs correlated with the clinical outcomes of tumors. Hence, these miRNAs could play potential role as prognostic biomarkers of cancers [10, 11]. Among them, miR-210 may be one of the most attracive biomarkers. Many studies have investigated that several miR-210 targets were more specifically in the context of cancer, including cell-cycle regulator E2F3, homeobox proteins (HOXA1, HOXA9) [12], and the iron sulfur cluster sssembly proteins ISCU1/2 [13], which are involved in many cellular processes such as heme biosynthesis and iron metabolism. Furthermore, it has been shown that depending on the tissue type or cellular model, miR-210 was able to inhibit apoptosis [14, 15] or to repress tumor initiation [12]. In clinical studies, miR-210 has been found to be associated with poor prognosis in early breast cancer in which miR-210 levels above the median are associated with lower survival at 10 years [16]. Additional studies demonstrated elevated miR-210 expression in other carcinomas, such as pancreatic cancer, glioblastoma, head and neck cancer, and renal cancer [17–20]. Therefore, abundant miR-210 may be a general feature of carcinoma and be used as a biomarker. However, contradictory data exist concerning the regulation and roles of miR-210 during cancer progression as miR-210 appears to be absent in ovarian carcinoma [21]. Thus, the expression of miR-210 in cancers and the prognostic significance remain unclear.

In this study, we conducted a systematic review and meta-analysis to summarize the findings globally for the use of miR-210 to predict the clinical results of cancer patients. And we also want to evaluate the overall risk of elevated miR-210 for survival in patients with cancers.

## 2. Materials and Methods

### 2.1. Search Strategy

We carefully searched online PubMed from 1966 to 7 March 2013 to identify relevant studies. The following strategies were used to retrieve articles and abstracts in English (microRNA-210 OR mir-210) AND (tumor OR neoplasm OR cancer).

### 2.2. Study Inclusion/Exclusion Criteria

Studies were considered eligible if they met all of the following inclusion criteria: (i) studied patients with any type of carcinoma; (ii) measured the expression of miR-210 in tissue or serum; and (iii) investigated the association between miR-210 expression levels and survival outcome. Articles were excluded based on the following criteria: (i) review articles, laboratory articles, or letters, (ii) articles that described the survival outcome of other markers, (iii) absence of key information for analysis with methods developed by Parmar et al. [22], Williamson et al. [23], and Tierney et al. [24], and (iv) articles from one author and the studies brought into the repeated samples from the same patients.

### 2.3. Data Extraction

Eligible articles were reviewed independently by two investigators (Minmin Li and Xuelei Ma) and disagreements were resolved by consensus. The simplest method consisted in the direct collection of HR and their 95% CI from the original article. If not available, we extracted univariate Cox hazard regression analysis or log-rank *P* value and Kaplan-Meier survival curves of survival outcomes instead. Additional data obtained from the studies included the following: first author, publication year, study size, patients' age, TNM stage, tumor size, sampling site, histological classification, methods to detect miR-210, follow-up, and the attitude conclusion.

### 2.4. Statistical Analysis

MiR-210 was considered positive or negative according to the cutoff values provided by the authors. Log Hazard Ratio (logHR) and standard error (SE) were statistically combined for the quantitative aggregation of the survival results, but the two statistical variables were not given explicitly in all of the studies. Therefore, based on methods developed by Parmar, Williamson, and Tierney, we calculated the logHR and SE with data as mentioned in “data extraction:” HR and their 95% CI, log-rank *P* value, or Kaplan-Meier survival curves.

A test of heterogeneity of combined HRs was carried out using Cochran's *Q* test and Higgins I-squared statistic. Heterogeneity was defined as *P* < 0.05 or *I* ^2^ > 50%. A fixed effect model was used in the absence of between-study heterogeneity (*P* ≥ 0.05, *I* ^2^ ≤ 50%), while the random effect model was applied if heterogeneity was observed (*P* < 0.05, *I* ^2^ > 50%). An observed HR > 1 indicated worse outcome for the positive group relative to the negative group, and if their 95% CI did not overlap 1, statistical significance would be considered. Publication bias was evaluated using the funnel plot with the Begg's rank correlation; *P* > 0.05 was considered that there was no potential publication bias. The publication bias is a major concern for all kinds of meta-analysis, because positive results tend to be accepted by journals, while negative ones are often rejected or even uncommitted. All above analyses were performed using RevMan 5.1 (Cochrane collaboration, Oxford, UK) and Publication biases were calculated using the Begg's funnel plot by STATA 11.0 (STATA Corporation, College Station, TX, USA).

## 3. Results

### 3.1. Eligible Studies

**Figure 1:**
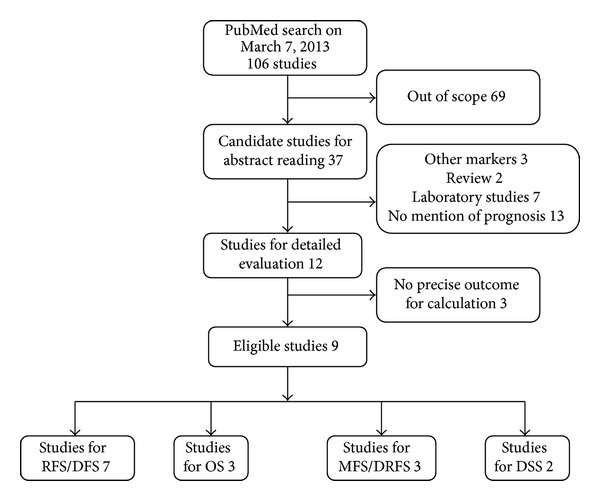
Selection of studies.

One hundred and six records for miR-210 and cancer were identified from a primary literature search in PubMed. The initial search yielded 69 studies and only 37 studies remained for candidate. Then we screened by titles, abstracts, and key words; 25 studies were excluded because 3 studies investigated other miRNAs but not miR-210; 13 studies did not deal with miR-210 expression data as a prognostic relevant variable; 7 studies were laboratory studies and the other 2 studies were review articles. Upon further full-text review of 12 studies, 3 studies were eliminated due to inadequate data for meta-analysis. Therefore, the final meta-analysis was carried out for the remaining 9 studies (Figure 1). Given that several authors have multiple publications in this field, we took great care to ensure that data reported in each paper were unique. If one study referred to a different subtype or provided survival data for both outcomes, which may include disease free survival (DFS), recurrence free survival (RFS), overall survival (OS), disease specific survival (DSS), metastasis free survival (MFS), or distant relapse free survival (DRFS), the study was listed twice. Considering the correlation of the two survival outcomes, we combined RFS and DFS together as RFS/DFS, while we combined MFS and DRFS together as MFS/DRFS.

The main features of eligible studies, published from 2008 to 2012, are summarized in Table 1. We collected data from 9 studies including a total of 1238 participants, with a median followup of 64.6 months (range: 9.4–120), from UK, Netherlands, Germany, USA, Italy, Columbia, and Japan. The patients were of a variety of carcinomas, including breast cancer, head and neck cancer, soft-tissue sarcoma, pancreatic cancer, and renal cell carcinoma. Of all the studies, 5 studies (*n* = 947) recruited breast cancer patients; for the remaining 4 studies, each referred to one type of tumor as mentioned previously. Quantitative real-time PCR (qRT-PCR) was the method used for miR-210 expression assessment and the samples were all cancerous tissues. Notably, the cutoff values of miR-210 were different in each study, with median applied in 8 studies and quartiles used in 1 study only.

### 3.2. Correlation between miR-210 Expression and Survival Outcome (RFS/DFS, OS, MFS/DRFS, and DSS)

#### 3.2.1. Overall Analysis

**Figure 2:**
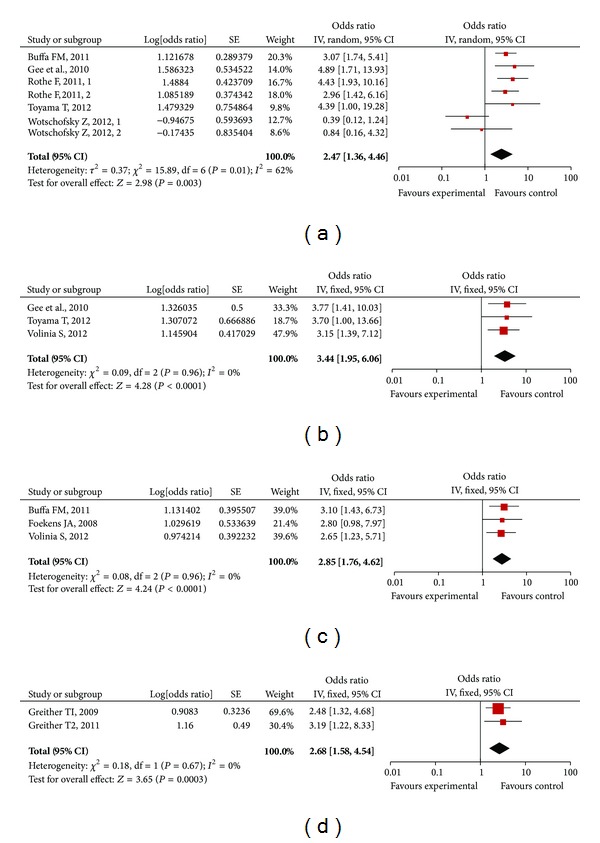
Forrest plots of studies evaluating hazard ratios of high miR-210 expression as compared to low expression in general carcinomas. Survival data are reported as recurrence free survival (RFS) or disease free survival (DFS) (a), overall survival (OS) (b), metastasis free survival (MFS) or distant relapse free survival (DRFS) (c), and disease specific survival (DSS) (d).

The main meta-analyses results are shown in Table 2 and Figure 2. In total, there were seven RFS/DFS studies, three OS studies, three MFS/DRFS studies, and two DSS studies.

For studies evaluating RFS/DFS, there appeared to be evidence for heterogeneity between HRs of miR-210 as assessed by inspection of Forrest plots (*n* = 699, *I* ^2^ = 62%, and *P* = 0.01). Hence, a random model was applied to calculate a pooled HR and its 95% CI. We found that higher expression levels of miR-210 significantly predicted poorer RFS/DFS, with the pooled HR (95% CI) being 2.47 [1.36, 4.46] (Figure 2(a)).The pooled HR was more significantly predictive than a single HR of each study. For each study, the *P* value varied from 0.0001 to 0.83, and three studies (Toyama T; Wotschofsky Z1; Wotschofsky Z2) had a *P* value ≥ 0.05, which was not statistically significant.

For studies evaluating the remaining three survival outcomes, fixed models were applied because they appeared to have homogeneity among studies. Increased miR-210 expression was also significantly correlated with OS, MFS/DRFS, and DSS, with the combined HR (95% CI), respectively, being: OS, 3.44 [1.95, 6.06] (*n* = 314, *I* ^2^ = 0%, *P* = 0.96) (Figure 2(b)); MFS/DRFS, 2.85 [1.76, 4.62] (*n* = 624, *I* ^2^ = 0%, and *P* = 0.96) (Figure 2(c)); DSS, 2.68 [1.58, 4.54] (*n* = 134, *I* ^2^ = 0%, and *P* = 0.67) (Figure 2(d)). The pooled HR of OS was more reliable because one of three studies had a single *P* value = 0.05 (95% CI: 1.00–13.66). Similarly, the pooled HR of MFS/DRFS had more predictive significance because one of three studies had a single *P* value = 0.05 (95% CI: 0.98–7.97).

#### 3.2.2. Subgroup Analysis

**Figure 3:**
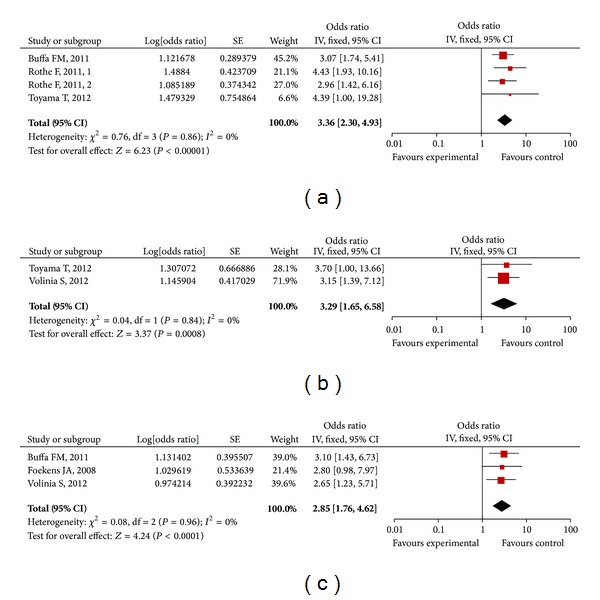
Forrest plots of studies evaluating hazard ratios of high miR-210 expression as compared to low expression in breast cancers. Survival data are reported as recurrence free survival (RFS) or disease free survival (DFS) (a), overall survival (OS) (b), and metastasis free survival (MFS) or distant relapse free survival (DRFS) (c).

When we grouped the meta-analyses by the tumor's type, we found that heterogeneity about RFS/DFS was not existed in studies of breast cancer any more (Table 2), which, however, existed in all of studies. According to above subgroup analysis, we hypothesized that tumor's type could be a main source of heterogeneity in the forest analysis. Importantly, elevated miR-210 level was still associated with worse survival outcome in patients with breast cancer, with the combined HR (95% CI) of RFS/DFS being 3.36 [2.30, 4.93] (*n* = 542, *I* ^2^ = 0%, and *P* = 0.86) (Figure 3(a)), HR (95% CI) of OS being 3.29 [1.65, 6.58] (*n* = 268, *I* ^2^ = 0%, and *P* = 0.84) (Figure 3(b)),and HR (95% CI) of MFS/DRFS being 2.85 [1.76, 4.62] (*n* = 624, *I* ^2^ = 0%, and *P* = 0.96) (Figure 3(c)), respectively.

The same results were shown in head and neck cancer, soft-tissue sarcoma, and pancreatic cancer (Table 2), even though only one study was relevant with the three types of tumors, respectively. However, the combined HR of RFS/DFS in renal cell carcinoma patients gave no clinical significance (Table 2).

### 3.3. Assessment of Publication Bias

**Figure 4:**
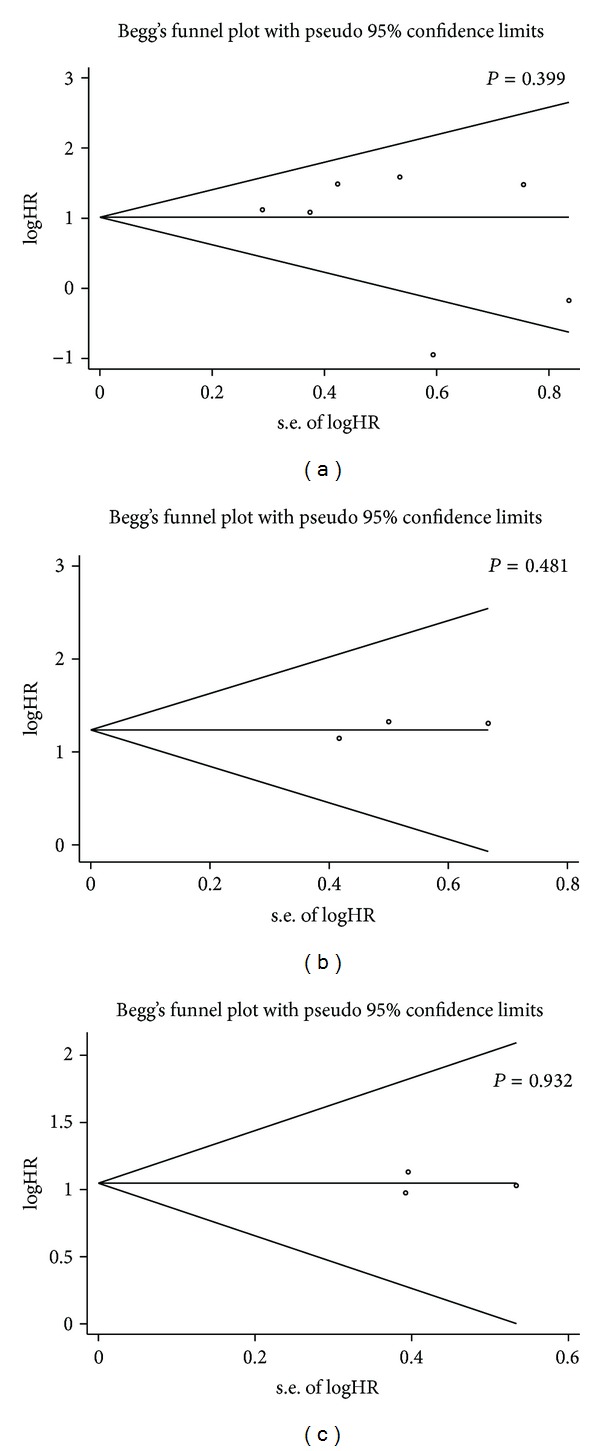
Funnel plots of publication bias summary for corresponding meta-analyses in Figure 2: (a) recurrence free survival (RFS) or disease free survival (DFS); (b) overall survival (OS); (c) metastasis free survival (MFS) or distant relapse free survival (DRFS).

Publication bias of the included studies was evaluated by funnel plots and Begg's test. As shown in Figure 4, in RFS/DFS, OS, and MFS/DRFS meta-analysis, the *P* values of Begg's regression intercepts were 0.399, 0.481, and 0.932, respectively. Hence, there was no significant publication bias in the meta-analysis, because their *P* values were not < 0.05. Since only two studies focused on DSS, we could not calculate its publication bias.

## 4. Discussion

Any insight into the future health of an individual patient with cancer is advantageous, and so efforts have been invested in figuring out reliable and informative evidence identifying prognostic biomarkers for patients with cancer in order to guide clinical decision making. During the last decade, miRNAs have been considered as potential biomarkers for cancer prognosis because they have unique expression profiles in cancerous tissue or serum compared to normal one, they have more stable expression than mRNA and they can be easily assessed by qRT-PCR [11].

Among these available miRNAs, miR-210 has been a striking one. The current meta-analysis, for the first time, confirmed that elevated miR-210 expression is a fine prognostic factor in patients with a variety of carcinomas. In our study, the pooled risk of miR-210 for RFS/DFS, OS, MFS/DRFS, or DSS in general cancers was not only statistically significant, but also strong, with combined HRs of 2.47, 3.44, 2.85, and 2.68, respectively. Empirically, HR > 2 is considered strongly predictive [25]. Except the combined HR of RFS/DFS for renal cell carcinoma group which was 0.50 [0.19, 1.30], the subgroup analyses grouped by the tumor's type were consistent with the overall analysis, especially in breast cancer. It may suggest that detected miR-210 expression in patients with cancers could predict their prognosis practically.

However, our conclusions should be tempered for several reasons. First, the numbers of prognostic studies dealing with each type of cancers, except for breast cancer, were < 5. Meanwhile, the results were less powerful because only 3 studies, respectively, were included for OS and MFS/DRFS meta-analyses, and only 2 studies were included for DSS. Second, because authors did not make a clearly defined cutoff of miR-210 in original articles, that made us unable to provide a clear clue about how high is high. Third, although this meta-analysis has revealed miR-210 as a prognostic biomarker, only one study mentioned it as an independent predictor. Lack of individual HR data of other markers makes it difficult to exclude the influences by confounding factors in a meta-analysis. The current statistical analysis could not deduce miR-210 an independent predictor due to methodological limitations.

Further, the risks calculated in our meta-analysis may be an overestimate as a result of several limits in our meta-analysis. First, heterogeneity was found in the meta-analysis for RFS/DFS of the prognostic role of miR-210 (*I* ^2^ = 62%, *P* = 0.01). The heterogeneity mainly came from the Toyama T' study and Wotschofsky Z' studies. These studies had opposite survival outcomes. When we removed them, the adjusted HR was 3.47 [2.39, 5.01] (*I* ^2^ = 0%, *P* = 0.78). Also, many other causes should be responsible for the heterogeneity, such as the disease type, the cutoff value of miR-210, the duration of followup, and others. When we stratified them according to tumor type, heterogeneity disappeared in breast cancer subgroup (*P* > 0.05). In order to minimize the effect of the heterogeneity on RFS/DFS, we used a random effect model. The publication bias was another problem for the meta-analysis. We attempted to minimize publication bias by making the literature search as complete as possible, using PubMed database. However, there were still some factors that may introduce bias. For instance, positive results tend to be accepted by journals, unpublished papers and abstracts were excluded because the required data were not available, and three studies were not included in the meta-analysis due to a lack of available, or calculated, survival data by analysis. Nevertheless, the Begg's test showed no significant publication bias in this study (*P* > 0.05), suggesting that the statistics obtained approximate the actual results.

The association between miR-210 expression and cancer outcome may be partly caused by its complicated relationship with hypoxia [26], which is an important mechanism of treatment resistance in various carcinomas and a frequent feature of poor-prognosis tumors. Recent studies have identified miR-210 in a microarray analysis as the predominant miRNA induced by hypoxia in cancer cell lines, such as breast, head and neck, lung, colon, and renal cancer cell lines [27], and demonstrated the direct regulatory role of hypoxia-inducible factor-1 alpha (HIF-1a) in its transcription [12, 16, 21]. Meanwhile, researchers had found that miR-210 had a relationship with keratinocyte proliferation in a murine model of ischemic wounds [28] and in cerebral ischemia [29]. Given that angiogenesis is another notable feature of poor-prognosis tumors and the role of hypoxia in angiogenesis induction, it is not surprising that miR-210 seems to affect tumors by angiogenesis.

Though some clinical studies and reviews have proven the application of the microRNAs [30–32] and our study strongly suggested potential use of miR-210 in predicts the survival outcomes of patients with cancers, several points should be considered about its clinical application. First, a clear definition should be made about the cutoff value of miR-210 level for increased survival risk. In the original articles we studied for this meta-analysis, researchers used median or quartiles value as the cutoff value, so the accurate value was different. Lack of abundant miR-210 expression data in global population makes it difficult to set a standard cutoff. Second, could miR-210 predict the survival outcomes of patients with cancers as a single factor, or should it be applied with a set of miRNAs? Recently, researchers have considered using a set of miRNAs in place of a single miRNA to increase the prediction power. A panel of miRNAs may be a stronger predictor for survival than a single miR-210. Third, could we use miR-210 in plasma/serum to replace tissue? Though all the articles in this meta-analysis used tumor tissues for miR-210 study, circulating markers are more acceptable because they can be assayed easier technically and be monitored throughout the life. Eun-Jung et al. [33] has shown that plasma miR-210 may be used to predict and perhaps monitor response to therapies that contain trastuzumab. More studies should be conducted to evaluate the prognostic value of miR-210 level in plasma/serum. For routine clinical application in the future, the above-mentioned problems need further study.

In conclusion, the meta-analysis suggested that the positive expression of tissue miR-210 is significantly associated with poor prognosis of various types of carcinoma all around the world, especially in patients with breast cancer, regardless of RFS/DFS, OS, or MFS/DRFS. These results should be confirmed by adequate multicenter designed prospective studies in future and more clinical investigations should be conducted before miR-210 can be implemented in the routine clinical management of cancer.

## Figures and Tables

**Table 1:** Characteristics of all identified studies.

Author	Country	*N*	Age (median)	Tumor grade (I.II/III.IV)	Tumor size (<=cm/>, unclear)	Sampling site	Tumor type	Histological type	Histological differentiation (G1/G2, G3, and unknown)	Method	Attitude	Follow-up time (median)	Survival outcome (OS, DFS)	Cutoff	MicroRNA type
Buffa FM, 2011	UK	219	55	NR	2.3 (median)	Tissue	Breast cancer	Ductal, lobular, mixed, and others	129, 65, and 25	qRT-PCR	Neg	10 y	DFRS, RFS	Median	miR-128a, -342, -27b, -150
Foekens JA, 2008	The Netherlands	298	52	NR	2 cm, 141/157	Tissue	Breast cancer	ER+/LNN, triple-negative	44, 161, and 93	qRT-PCR	Pos	99 m	MFS	Median	miR-7, -128a, and -516-3p
Gee et al., 2010 [19]	UK	46	63	8, 38	NR	Tissue	Head and neck cancers	HNSCC	27, 19	qRT-PCR	Pos	41 m	OS, RFS	Median	miR-21, -10b
Greithe T1, 2009	Germany	56	61.7	NR	NR	Tissue	Pancreatic cancer	PDAC	NR	qRT-PCR	Pos	15.99 m	DSS	Median	miR-155, -203, -216, -217, and -222
Greithe T2, 2011	Germany	78	57	44, 34	NR	Tissue	Soft-tissue sarcoma	STS, LS, MFH, FS, RMS, LMS, NS, and Syn	48, 30	qRT-PCR	Pos	66 m	DSS	Quartiles	
Rothe F, 2011	USA	73	NR	NR	2 cm, 43/29/1	Tissue	Breast cancer	ER-positive, ER-negative	35, 22, and 16	qRT-PCR	Pos	9.23 y	RFS	Median	miR-148a
		89	NR	NR	2 cm, 34/55	Tissue	Breast cancer	ER-positive	64, 17, and 8	qRT-PCR	Pos	7.12 y	RFS	Median	miR-149a
Toyama T, 2012	Japan	161	NR	NR	2 cm, 42/117/2	Tissue	Breast cancer	TNBC, ER-positive, HER2-negative	86, 53, and 22	qRT-PCR	Pos	5.4 y	DFS, OS	Median	
Volinia S, 2012	Italy, Columbia	107	NR	NR	NR	Tissue	Breast cancer	DCIS, IDC, metaplastic, atypical, medullary, apocrine, adenoid, normal	NR	qRT-PCR	Pos	NR	MFS, OS	Median	miR-127-3p, -185, -143, -let-7b, -21, -221, -652, -106b, -197, and -let-7i
Wotschofsky Z, 2012	Germany	89	65	55, 34	5 (median)	Tissue	Renal cell carcinoma	ccRCC nonmetastatic	81, 8	qRT-PCR	Neg	33.8 m	RFS	Median	miR-122, -141, -155, -184, -200c, -224, and -514
		22	62	3, 19	9 (median)	Tissue	Renal cell carcinoma	ccRCC metastatic	8, 14	qRT-PCR	Neg	9.4 m	RFS	Median	miR-122, -141, -155, -184, -200c, -224, and -515

NR: not reference; qRT-PCR: quantitative reverse transcription-polymerase chain reaction; Pos: positive; Neg: negative; HNSCC: head and neck squamous cell carcinoma; PDAC: pancreatic ductal adenocarcinomas; STS: soft-tissue sarcoma; LS: liposarcoma; MFH: malignant fibrous histiocytoma; FS: fibrosarcoma; RMS: rhabdomyosarcoma; LMS: leiomyosarcoma; NS: neurogenic sarcoma; Syn: synovial sarcoma; TNBC: triple- negative breast cancers; ccRCC: clear cell renal cell carcinomas; G1, G2, and G3: histological different grades; DFRS: distant relapse-free survival; RFS: recurrence free survival; MFS: metastasis free survival; OS: overall survival; DSS: disease specific survival; DFS: disease free survival.

**Table 2:** Meta-analyses of miR-210 expression to predict the survival outcome.

	Survival outcome	Study *n*.	Patient *n*.	Model	HR (95% CI)	*P* value	Heterogeneity (*I* ^2^, *P*)	Conclusion
miR-210 total	RFS/DFS	7	699	Random	2.47 [1.36, 4.46]	0.003	62%, 0.01	Positive
	OS	3	314	Fixed	3.44 [1.95, 6.06]	<0.0001	0%, 0.96	Positive
	MFS/DRFS	3	624	Fixed	2.85 [1.76, 4.62]	<0.0001	0%, 0.96	Positive
	DSS	2	134	Fixed	2.68 [1.58, 4.54]	0.0003	0%, 0.67	Positive
miR-210 breast cancer	RFS/DFS	4	542	Fixed	3.36 [2.30, 4.93]	<0.00001	0%, 0.86	Positive
	OS	2	268	Fixed	3.29 [1.65, 6.58]	0.0008	0%, 0.84	Positive
	MFS/DRFS	3	624	Fixed	2.85 [1.76, 4.62]	<0.0001	0%, 0.96	Positive
miR-210 head and neck cancer	RFS/DFS	1	46	Fixed	4.89 [1.71, 13.93]	0.003	—	Positive
	OS	1	46	Fixed	3.77 [1.41, 10.03]	0.008	—	Positive
miR-210 soft-tissue sarcoma	DSS	1	78	Fixed	3.19 [1.22, 8.33]	0.02	—	Positive
miR-210 pancreatic cancer	DSS	1	56	Fixed	2.48 [1.32, 4.68]	0.005	—	Positive
miR-210 renal cell carcinoma	RFS/DFS	2	111	Fixed	0.50 [0.19, 1.30]	0.16	0%, 0.45	Negative

## References

[1] Lee RC, Feinbaum RL, Ambros V (1993). The C. elegans heterochronic gene *lin-4* encodes small RNAs with antisense complementarity to *lin-14*. *Cell*.

[2] Filipowicz W, Bhattacharyya SN, Sonenberg N (2008). Mechanisms of post-transcriptional regulation by microRNAs: are the answers in sight?. *Nature Reviews Genetics*.

[3] Bartel DP (2004). MicroRNAs: genomics, biogenesis, mechanism, and function. *Cell*.

[4] Calin GA, Dumitru CD, Shimizu M (2002). Frequent deletions and down-regulation of micro-RNA genes *miR15* and *miR16* at 13q14 in chronic lymphocytic leukemia. *Proceedings of the National Academy of Sciences of the United States of America*.

[5] Esquela-Kerscher A, Slack FJ (2006). Oncomirs—microRNAs with a role in cancer. *Nature Reviews Cancer*.

[6] Bushati N, Cohen SM (2007). MicroRNA functions. *Annual Review of Cell and Developmental Biology*.

[7] Croce CM (2009). Causes and consequences of microRNA dysregulation in cancer. *Nature Reviews Genetics*.

[8] Calin GA, Croce CM (2006). MicroRNA signatures in human cancers. *Nature Reviews Cancer*.

[9] Iorio MV, Ferracin M, Liu C-G (2005). MicroRNA gene expression deregulation in human breast cancer. *Cancer Research*.

[10] Nana-Sinkam P, Croce CM (2010). MicroRNAs in diagnosis and prognosis in cancer: what does the future hold?. *Pharmacogenomics*.

[11] Ferracin M, Veronese A, Negrini M (2010). Micromarkers: miRNAs in cancer diagnosis and prognosis. *Expert Review of Molecular Diagnostics*.

[12] Huang X, Ding L, Bennewith KL (2009). Hypoxia-inducible mir-210 regulates normoxic gene expression involved in tumor initiation. *Molecular Cell*.

[13] Chan SY, Zhang Y-Y, Hemann C, Mahoney CE, Zweier JL, Loscalzo J (2009). MicroRNA-210 controls mitochondrial metabolism during hypoxia by repressing the iron-sulfur cluster assembly proteins ISCU1/2. *Cell Metabolism*.

[14] Kulshreshtha R, Ferracin M, Wojcik SE (2007). A microRNA signature of hypoxia. *Molecular and Cellular Biology*.

[15] Fasanaro P, D’Alessandra Y, di Stefano V (2008). MicroRNA-210 modulates endothelial cell response to hypoxia and inhibits the receptor tyrosine kinase ligand ephrin-A3. *The Journal of Biological Chemistry*.

[16] Camps C, Buffa FM, Colella S (2008). hsa-miR-210 is induced by hypoxia and is an independent prognostic factor in breast cancer. *Clinical Cancer Research*.

[17] Greither T, Grochola LF, Udelnow A, Lautenschläger C, Würl P, Taubert H (2010). Elevated expression of microRNAs 155, 203, 210 and 222 in pancreatic tumors is associated with poorer survival. *International Journal of Cancer*.

[18] Shuwei Q, Sheng L, Dan H (2013). Interactions of miR-323/miR-326/miR-329and miR-130a/miR-155/miR-210 as prognostic indicators for clinical outcome of glioblastoma patients. *Journal of Translational Medicine*.

[19] Gee HE, Camps C, Buffa FM (2010). hsa-miR-210 is a marker of tumor hypoxia and a prognostic factor in head and neck cancer. *Cancer*.

[20] Neal CS, Michael MZ, Rawlings LH, van der Hoek MB, Gleadle JM (2010). The VHL-dependent regulation of microRNAs in renal cancer. *BMC Medicine*.

[21] Giannakakis A, Sandaltzopoulos R, Greshock J (2008). miR-210 links hypoxia with cell cycle regulation and is deleted in human epithelial ovarian cancer. *Cancer Biology and Therapy*.

[22] Parmar MK, Torri V, Stewart L (1998). Extracting summary statistics to perform meta-analyses of the published literature for survival endpoints. *Statistics in Medicine*.

[23] Williamson PR, Smith CT, Hutton JL, Marson AG (2002). Aggregate data meta-analysis with time-to-event outcomes. *Statistics in Medicine*.

[24] Tierney JF, Stewart LA, Ghersi D, Burdett S, Sydes MR (2007). Practical methods for incorporating summary time-to-event data into meta-analysis. *Trials*.

[25] Hayes DF, Isaacs C, Stearns V (2001). Prognostic factors in breast cancer: current and new predictors of metastasis. *Journal of Mammary Gland Biology and Neoplasia*.

[26] Huang X, Le Q-T, Giaccia AJ (2010). miR-210—micromanager of the hypoxia pathway. *Trends in Molecular Medicine*.

[27] Kulshreshtha R, Ferracin M, Wojcik SE (2007). A microRNA signature of hypoxia. *Molecular and Cellular Biology*.

[28] Biswas S, Roy S, Banerjee J (2010). Hypoxia inducible microRNA 210 attenuates keratinocyte proliferation and impairs closure in a murine model of ischemic wounds. *Proceedings of the National Academy of Sciences of the United States of America*.

[29] Yuan-Lei L, Fei G, Fen L (2012). miR-210 activates notch signaling pathway in angiogenesis induced by cerebral ischemia. *Molecular and Cellular Biochemistry*.

[30] Liang Y (2008). An expression meta-analysis of predicted microRNA targets identifies a diagnostic signature for lung cancer. *BMC Medical Genomics*.

[31] de Planell-Saguer M, Rodicio MC (2011). Analytical aspects of microRNA in diagnostics: a review. *Analytica Chimica Acta*.

[32] Wei J, Gao W, Zhu C-J (2011). Identification of plasma microRNA-21 as a biomarker for early detection and chemosensitivity of non-small cell lung cancer. *Chinese Journal of Cancer*.

[33] Eun-Jung J, Libero S, Juyeon K (2012). Plasma microRNA 210 levels correlate with sensitivity to trastuzumab and tumor presence in breast cancer patients. *Cancer*.

